# Stress drives a switch in sex preference

**DOI:** 10.1126/science.adu7946

**Published:** 2025-01-09

**Authors:** Bitna Joo, Kay M. Tye

**Affiliations:** 1 Salk Institute for Biological Studies, La Jolla, CA, USA.; 2 University of California San Diego, La Jolla, CA, USA.; 3 Howard Hughes at Salk Institute, La Jolla, CA, USA.; 4 Howard Hughes Investigator and Wylie Vale Professor at Salk Institute, La Jolla, CA, USA.; 5 Kavli Institute for the Brain and Mind, La Jolla, CA, USA. Author.

## Abstract

Distinct brain circuits support sex preferences in mice

To date, much of the focus in social neurobiology research has been on characterizing the motivational importance of a social stimulus. For example, how an encounter with another individual presents a threat or a potential mate? However, understanding the absolute (fixed) features of a social stimulus has lagged. On page 155 of this issue, Wei *et al*. (1) report the distinct sexually dimorphic neural circuits that encode a switch in absolute sexual preference in mice that allow both sexes to prefer interacting with females under normal conditions, but swap to preferring male interactions when exposed to threatening stimuli. The findings point to a flexible control mechanism shared by male and female animals for social interactions.

When animals navigate the physical landscape, two types of spatial representations are used--allocentric cognitive maps that are based on the absolute spatial relationships between objects in the environment, and egocentric cognitive maps that are centered around the navigator’s relative position and orientation (2). Analogous to this, animals may also navigate the social landscape using relative or absolute maps. Although the neural mechanisms underpinning how animals achieve this are poorly understood, multiple circuits are involved in determining how the brain evaluates and responds to the emotional importance of stimulus(processing positive or negative valence) (3), whereas motivational importance can be shifted by context, internal state, or prior experiences.

Wei *et al*. sought to investigate the neural mechanisms underlying social preference for male or female interactions in mice. The authors operationalized sex preference as a concept distinct from that of sexual orientation, describing what other mice a given mouse wants to be near rather than what other mice it is sexually attracted to. As with physical navigation, sex preference can be framed in relative or absolute terms. For example, a mouse may identify another mouse of the opposite sex as a mate, in relative terms. At the same time, a mouse can be male in absolute terms, regardless of who we are (e.g. sex, status, age). In humans, these two frameworks exist seamlessly in the mind, and the psychology of sex preference, identity, and orientation has rich, rapidly-evolving literature and vocabulary to navigate between relative and absolute paradigms.

Social preference in some animals can be influenced by absolute characteristics (e.g. size, strength) and relative ones, such as familiarity, social hierarchy, and environmental contexts (e.g. threat or safety). Wei *et al*. uncovered a fascinating phenomenon wherein the presentation of a threat-associated stimulus shifts the social preference from females to males for both sexes – a universal shift in absolute sex preference. Specifically, they found that both male and female mice prefer to socialize with female mice at baseline (normal conditions). However, when the authors presented mice with innate threats such as predator odor (2,4,5-trimethylthiazoline), they observed a shift in preference to male mice (or male bedding) over female mice (or female bedding) for both sexes. This phenomenon also applied to learned threats, such as shock-paired cue in which a neutral stimulus (a cue) becomes associated with a stimulus (a shock) through repeated pairing, leading to a conditioned response (fear) when the cue is presented alone. Notably, sex preferences were measured by time spent with both male and female mice, or bedding from male or female cages, which allowed for rigorous discrimination of representations of sex preference rather than of sexually-dimorphic movement patterns, such as those associated with mating (4).

To explore what neuronal circuitry accounts or this switch in sexual preference, Wei *et al*. used dual-color fiber photometry recordings of neuronal activity and projection-specific chemogenetic/optogenetic manipulation of dopamine (DA) neurons in the ventral tegmental area (VTA) of the mouse brain to identify distinct projections of ventral tagmental area dopamine (VTA^DA^) neuron activity. They found that the sexually dimorphic effect was caused by specific changes in the projections from the VTA to the nucleus accumbens (NAc) or the medial preoptic area of the hypothalamus (mPOA). The NAc is referred to as the brain’s reward center because it processes pleasure, reward, and motivation. The mPOA has been implicated in animal movement associated with mating in mice. Wei et al. observed that in male mice, the VTA^DA^-mPOA circuit mediates male preference whereas the VTA^DA^-NAc projection regulates female preference. By contrast, in female mice, sex preference is regulated solely by the VTA^DA^-NAc projection: steady, regular (tonic) firing of neurons drives male preference, whereas firing in bursts (phasic) promotes female preference (see the figure).

Notably, the universal expression of sex preferences across males and females requires an absolute representation, rather than a relational representation. Prior studies in mice that focused on VTA^DA^ neurons in social behavior have shown that these cells bidirectionally modulate social behavior—that is, they can either promote or suppress social behavior depending on stimulation or inhibition of neurons and that the activation of VTA-NAc circuit increases social interaction (5). However, these findings were based exclusively on studies in male mice, leaving a gap in understanding of how male and female differentially operate these mechanisms.

One possible explanation for the observations of Wei *et al*. is that the mPOA regulates mating and parenting behaviors. Optogenetic activation of mPOA induces male-typical mounting behavior as well as pup retrieval behavior in both sexes (6). A subset of neurons in the mPOA that secrete the neuropeptide galanin elicit both male and female parenting behavior, and activation of these neurons reduces aggression and increases pup grooming in males (7). In female mice, estrogen receptor alpha (Esr1)-expressing neurons in the mPOA (mPOA^Esr1^) mediate pup approach and retrieval, and the mPOA^Esr1^ to VTA projections drive maternal behaviors (6, 8).

Another possibility is that, given the role of the VTA-NAc circuit in counteracting stress-induced depressive behaviors in mice and rat (9, 10), the presentation of stressors could recruit this circuit. Phasic, not tonic, firing of VTA^DA^ neurons underlies place preference, condition in which the animal associates a particular environment with a rewarding experience (11). This phasic firing also rescues stress-induced depression-like phenotypes and escape-related behaviors (9). Furthermore, female hamsters find female social interactions more rewarding than do males, and receptors in the VTA for the hormone oxytocin regulate social reward in both sexes (12). These observations support the hypothesis that male and female mice may have different set points for social reward. For female mice, interactions with other females could exert a stress buffering, antidepressive effect, whereas encountering male mice might serve as a hedonic reward, signaled by phasic bursts of VTA^DA^ neurons.

The discovery that the same absolute sex preference is mediated by distinct neural circuits in male and female mice provides insight into how a universal shift in absolute sex preference can be shared by both sexes despite their distinct motivational importance for mate preference. Whether these same principles apply in humans, given their more complex representations of sex, sexuality, and preference, remains to be determined. Understanding the degree to which these biological mechanisms are conserved across species and the independent evolution of sociability calls for further investigation.

## Figures and Tables

**Figure.**
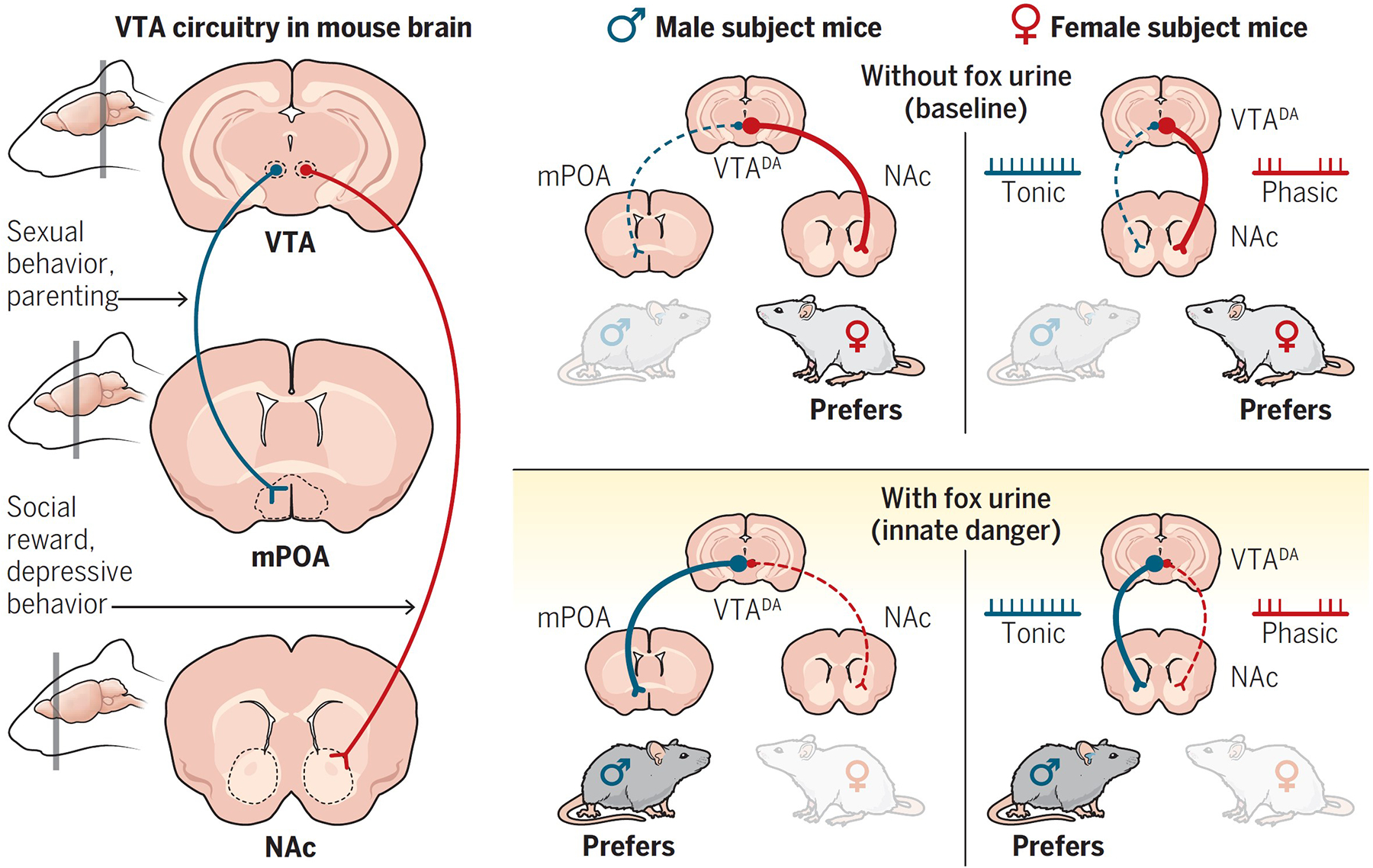
Sexually dimorphic VTA dopaminergic circuit mechanisms in male and female mice drive sex preference. A. Current understanding of VTA circuitry implicated in different social behaviors in mice. B. In males (left), VTA-NAc circuitry governs female preference without threat (upper left), while VTA-mPOA circuitry governs male preference in the presence of a threat signal (below left). By contrast, only the VTA-NAc circuitry shapes preference in females (right), with phasic firing driving female preference without threat (upper right) and tonic firing mediates male preference in the presence of a threat signal (below right). VTA, ventral tagmental area; NAc, nucleus accumbens; mPOA, medial preoptic area.
